# Summary and Conclusions: New Perspectives in Hereditary Angioedema: Molecular Mechanisms and Therapeutic Choices: *A CME Symposium Presented at the World Allergy Congress, Buenos Aires, Argentina (December 2009)*

**DOI:** 10.1097/1939-4551-3-S3-S39

**Published:** 2010-09-15

**Authors:** Bruce L Zuraw

**Affiliations:** 1 Department of Medicine, University of California San Diego and San Diego Veteran's Affairs Medical Center, La Jolla, CA

## 

Hereditary angioedema (HAE) is a relatively rare disease, with an estimated prevalence of approximately 1 per 50,000 in the general population. Despite its rarity, HAE has become an area of intense interest within the medical community. This interest likely reflects several different aspects of HAE, including: the high potential for significant morbidity and mortality associated with attacks of angioedema in HAE patients, the frequent failure to make an accurate and timely diagnosis, the progress in unraveling the underlying pathophysiology of HAE, and the tremendous advances in treatment options. To limit the morbidity and mortality that has long afflicted patients with HAE, it has become increasingly important that the clinician understand both the underlying mechanisms of swelling in HAE and the newer treatment strategies that have been developed for HAE. This supplement contains 3 reviews that summarize the current state-of-the-art regarding the pathophysiology and treatment of HAE.

The pathophysiology of HAE is reviewed by Zuraw in this supplement. The discovery that HAE patients were deficient in C1 inhibitor (C1INH) quickly led to the subsequent discovery that the protein could either be nonsecreted (Type I HAE) or dysfunctional (Type II HAE). These observations led to the cloning of the gene for C1INH (SERPING1), and the discovery that HAE ultimately results from a mutation of that gene. Type II HAE has been found to result almost exclusively from mutations on the C1INH reactive mobile loop, while Type I HAE results from mutations scattered throughout the gene.

C1INH inactivates a large number of plasma serine proteases, including the complement proteases and contact system proteases. In the absence of sufficient C1INH, these proteolytic cascades become dysregulated. An elegant series of experiments demonstrated that bradykinin, generated through activation of the plasma contact system, is the primary mediator of swelling in HAE. Once generated, bradykinin binds to bradykinin B2 receptors on vascular endothelial cells and increases vascular permeability primarily by disrupting the tight junctions that normally prevent water movement through the endothelial layer. Bradykinin catabolism is mediated by several peptidases, and there is evidence that variation in the level of these peptidases by drugs or genetic polymorphisms may influence bradykinin actions.

C1INH replacement therapy is reviewed by Frank in this supplement. Although several antifibrinolytics and anabolic androgen drugs can decrease HAE attack frequency and severity when administered prophylactically, their efficacy was not perfect and was further limited by side effects. While C1INH concentrates have been widely used to treat acute attacks of HAE for many years, there was limited randomized trial data to support this and these drugs were not available in the United States.

Three different C1INH concentrates were recently tested in double-blind randomized placebo-controlled studies. All 3 concentrates demonstrated significant benefit and excellent safety when used to treat acute attacks of HAE. A pasteurized plasma derived C1INH (pdC1INH, Berinert, CSL Behring) 20 U/kg was licensed for use in the treatment of acute attacks in the United States and is currently in use in Europe. A recombinant human C1INH (rhC1INH, Rhucin, Pharming) was recently approved in the European Union for this indication and should shortly be reviewed by the FDA. Nanofiltered pasteurized plasma derived C1INH (nf-C1INH, Cinryze, ViroPharma) did not receive approval for the acute indication in the United States. The nf-C1INH was also studied in a separate prophylactic trial and shown to significantly protect HAE patients against attacks when administered at a dose of 1000 U every 3-4 days. Based on this, nf-C1INH has been licensed in the United States for prophylactic use.

In addition to C1INH replacement, drugs targeting bradykinin generation and actions has been developed and are reviewed by Riedl in this supplement. A potent and specific plasma kallikrein inhibitor (ecallantide, Kalbitor, Dyax) was shown in 2 separate double-blind randomized placebo-controlled studies to be effective in treating acute attacks of HAE. Ecallantide is administered by subcutaneous injection at a dose of 30 mg and is licensed in the United States but not Europe for treatment of acute attacks.

A separate approach is to antagonize the action of bradykinin using a bradykinin B2 receptor antagonist. Icatibant (Firazyr, Shire) is a specific bradykinin B2 receptor antagonist that is administered by subcutaneous injection at a dose of 30 mg. Two double-blind randomized placebo-controlled studies assessed the efficacy of icatibant in the treatment of acute attacks. The European study successfully demonstrated the efficacy of icatibant, and it is now licensed in Europe for the treatment of acute attacks. The American study, however, failed to meet its primary end point. As a consequence, an additional trial is currently underway in the United States.

## Conclusions

Elucidation of the underlying pathophysiology of HAE directly led to the development of a number of new and effective drugs to treat this disease. Based on the available evidence, pdC1INH, nf-C1INH, rhC1INH, ecallantide, and icatibant all seem to be effective for the treatment of acute attacks in HAE. In addition, nf-C1INH appears to be effective for long-term prophylaxis of HAE. Understanding the safety profile of these drugs will require additional monitoring; however, all 5 seem to be generally well tolerated.

A key need for the future will be to better define the advantages and disadvantages of each of these drugs and develop algorithms to optimize the care of patients with HAE, both with respect to which patients should receive prophylactic treatment and which drug to use to treat acute attacks. HAE disease severity is highly variable, and treatment will need to be optimized based on individual patient needs. Ultimately, the success of these promising new drugs will be judged by the extent to which HAE patients can resume a normal life.

